# A Prediction Model for Uncoating Receptor Usage in Human Enteroviruses Based on Amino Acid Sequences and a Naive Bayes Algorithm

**DOI:** 10.3390/v18020236

**Published:** 2026-02-13

**Authors:** Yongtao Jia, Zhenyu Xie, Guoying Zhu, Changzheng Dong

**Affiliations:** 1 Jiaxing Center for Disease Control and Prevention, Jiaxing 314000, China; yongtao_jyt@163.com (Y.J.); jxcdcxiezhenyu@163.com (Z.X.); 2 School of Public Health, Health Science Center, Ningbo University, Ningbo 315211, China

**Keywords:** human enterovirus, uncoating receptor, amino acid sequences, bioinformatics prediction algorithm, machine learning

## Abstract

This study constructed a bioinformatics prediction algorithm for human enterovirus uncoating receptors based on amino acid sequences and physicochemical properties. Based on the availability of uncoating receptor information and three-dimensional (3D) structural data, human enterovirus serotypes were classified into training, validation, and prediction datasets. Using amino acid sequences of receptor-binding sites and their physicochemical properties as model features, a prediction model was constructed using the Naive Bayes algorithm and bioinformatic network analysis method. The results showed that both the training and validation datasets achieved a prediction accuracy of 100%. Among the 56 serotypes in the prediction dataset, the vast majority utilized seven known types of uncoating receptors (e.g., SCARB2, CAR, and ICAM-1), while a minority of serotypes may share the same novel, unknown receptor. This study indicates that uncoating receptors can be accurately predicted based on the amino acid sequences and physicochemical properties of human enteroviruses. Furthermore, the three-dimensional structural features at receptor-binding sites can be reflected through corresponding amino acid sequences and their physicochemical properties. This study facilitates a more in-depth investigations of enterovirus pathogenic mechanisms and provides important insights for the development of vaccines and antiviral drugs.

## 1. Introduction

Human enteroviruses, belonging to the genus Enterovirus within the family Picornaviridae, are classified into four species: Enterovirus A (EVA), Enterovirus B (EVB), Enterovirus C (EVC), and Enterovirus D (EVD). These viruses comprise more than 100 serotypes, including Enterovirus 71 (EV71), Coxsackieviruses (CVs), Echoviruses (Es), and Polioviruses (PVs). They cause a wide range of diseases, such as hand, foot, and mouth disease (HFMD), viral encephalitis and myocarditis, acute flaccid paralysis, and poliomyelitis, primarily imposing a significant disease burden on infants and young children [1,2,3,4]. Currently, except for vaccines targeting PV and EV71, no licensed vaccines are available for other enteroviruses.

**Figure 1:**
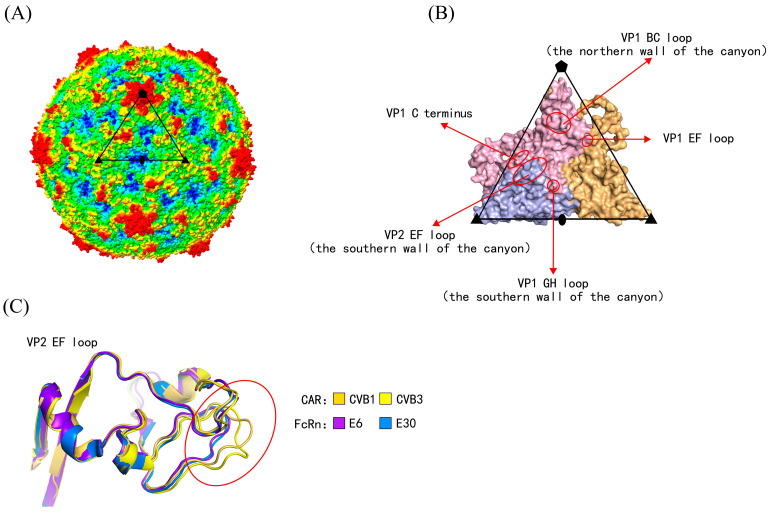
The capsid structures of human enterovirus. (**A**) Radius-colored capsid surface representation of human enterovirus. Based on the distance from the particle center, the capsid surface is colored from blue to red (red representing the furthest distance). The subunit is outlined in black. Pentagons, triangles, and ellipses represented the fivefold, threefold, and twofold axes, respectively. (**B**) Magnified views of the subunit structure. The secondary structures are marked by arrows and circles, forming the canyon. The receptor-binding sites are located on these secondary structures. VP1–VP3 are indicated in light pink, light blue, and light orange, respectively. (**C**) Serotypes utilizing different uncoating receptors exhibited three-dimensional structural differences at the secondary structure.

Human enteroviruses are a group of non-enveloped viruses with single-stranded, positive-sense RNA genomes. The RNA genomes are encapsulated within protective protein shells called the capsid. The capsid is composed of 60 asymmetric subunits, each consisting of four structural proteins, termed viral proteins (VPs) or capsid proteins (Figure 1A,B) [5]. VP1, VP2, and VP3 constitute the outer surface of the capsid, whereas VP4 is located on the interior. The capsid plays essential roles in host cell receptor binding, antibody-mediated neutralization, and interactions with antiviral drugs [6,7,8]. Adjacent to the fivefold axis of the capsid lies a canyon-like depression. The canyon is formed by the VP1 BC loop (constituting the northern wall of the canyon), the VP1 GH loop, and the VP2 EF loop (constituting the southern wall) [9]. The receptor-binding regions are located within the canyon (Figure 1B) [10].

Enterovirus infection is initiated by the binding of the viral particles to specific receptors on the host cell surface [8,11]. These host cell receptors are functionally categorized into two main types: uncoating receptors and attachment receptors [11]. Uncoating receptors induce conformational changes in viral particles, thereby facilitating the release of viral genomes from the capsid into the host cell cytoplasm. In contrast, attachment receptors assist uncoating receptors in accomplishing this process [12,13,14,15]. To date, seven uncoating receptors have been experimentally identified across different enterovirus species: EVA utilizes Scavenger receptor class B, member 2 (SCARB2) [14] and Kringle containing transmembrane protein 1 (KREMEN1) [16]; EVB utilizes Coxsackievirus and adenovirus receptor (CAR) [17,18] and Human neonatal Fc receptor (FcRn) [9,15]; EVC uses Intercellular adhesion molecule 1 (ICAM-1) [19,20] and Cluster of differentiation 155 (CD155), also known as Poliovirus receptor (PVR) [21]; and EVD utilizes ICAM-5 [22]. Except for the interaction between ICAM-5 and EVD, three-dimensional (3D) structures of enterovirus–receptor complexes for other species have been determined. These structures have provided high-resolution details of the receptor-binding regions on the viral capsid surfaces [9,14,15,16,17,18,19,20,21]. The utilization of uncoating receptors follows a pattern in which each serotype typically utilizes a specific receptor, while multiple serotypes share the same receptor [23,24]. Structurally, the receptor-binding regions for most uncoating receptors are located within the canyon on the viral capsid. A notable exception is SCARB2, whose binding region is positioned on the southern rim of the canyon [14]. However, the mechanisms by which receptor-binding regions determine receptor usage remain unclear. Additionally, the uncoating receptors for many enteroviruses have not yet been identified.

The antibody blocking assay is a classical method used to identify viral receptors [23]. However, it faces challenges in differentiating uncoating receptors from attachment receptors and has limited capability to identify novel unknown receptors [25]. Wang et al. [9] developed a clustering algorithm based on the 3D structures of receptor-binding regions in enteroviruses, which successfully predicted the uncoating receptors for 18 serotypes across three species (A/B/C). Nevertheless, this algorithm depends on viruses and receptors with available 3D structures, limiting its applicability to predicting receptors for a broader range of enteroviruses.

Based on the hypothesis that viral 3D structure is determined by its amino acid sequences and physicochemical properties, this study developed a bioinformatics prediction algorithm grounded in the amino acid sequences of enteroviruses. Using amino acid sequences and key physicochemical properties as model features, the algorithm employed the machine learning algorithm and bioinformatic network analysis method to predict enterovirus uncoating receptors. Compared with previous methods for identifying uncoating receptors, this study employed bioinformatic prediction algorithms instead of experimental approaches. Furthermore, it relied on readily available sequence data and physicochemical property information instead of the more difficult-to-obtain 3D structural data, thereby substantially improving the efficiency and practicality of uncoating receptor identification for enteroviruses.

This algorithm is applicable to enteroviruses with known amino acid sequences but unknown three-dimensional structures, covering a broader range of enteroviruses. Receptor usage determines cellular tropism and is closely associated with viral pathogenicity. Using the prediction algorithm, this study enables the rapid identification of uncoating receptor usage across human enterovirus serotypes. The algorithm provides guidance for investigating tissue tropism and pathogenic differences of serotypes in studies of enterovirus pathogenesis, predicts whether serotypes have the potential to invade specific tissues, pre-identifies the tissues most likely to be targeted, and provides clear directions for experimental design in studies of viral pathogenesis. This study further reveals the relationship between capsid structure and amino acid sequence in enteroviruses, and provides support for research on enterovirus pathogenic mechanisms, as well as the development of antiviral drugs and vaccines.

## 2. Materials and Methods

### 2.1. Construct Model Datasets and Serotype Pairs

**Figure 2:**
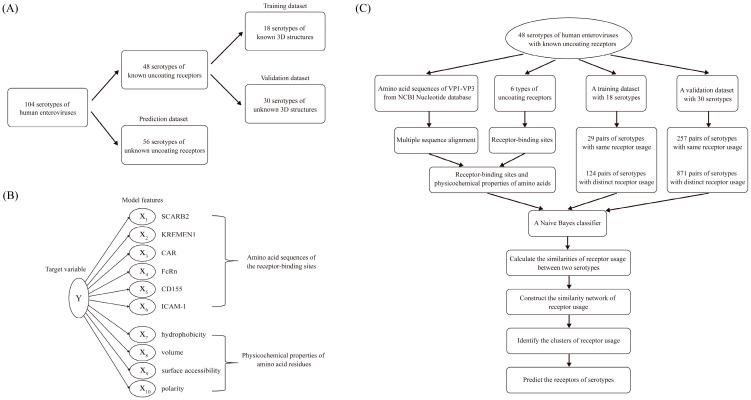
Bioinformatics prediction algorithm for human enterovirus uncoating receptors. (**A**) Construction of model datasets. A total of 104 serotypes with determined genomic sequences were classified into three datasets. The training dataset comprised 18 serotypes with known uncoating receptors and determined three-dimensional (3D) structures of viral capsids. The validation dataset comprised 30 serotypes with known uncoating receptors but undetermined 3D structures of viral capsids. The prediction dataset comprised 56 serotypes with unknown uncoating receptors and undetermined 3D structures of viral capsids. (**B**) Model feature selection. The receptor-binding sites of six uncoating receptors were determined, and four physicochemical properties of amino acids were selected due to their close association with the 3D structure of the viral capsid. (**C**) Flowchart of the prediction algorithm. The prediction algorithm consisted of two stages. In the first stage, a Bayesian method was used to calculate the receptor similarity between serotype pairs. In the second stage, receptor classification clusters were determined through bioinformatic network analysis.

Prototype strain sequences of human enterovirus serotypes were collected, and a total of 104 serotypes with determined genomic sequences were included. Based on the availability of uncoating receptor information and three-dimensional structural data, human enterovirus serotypes were categorized into training, validation, and prediction datasets (Figure 2A). The training dataset comprised 18 serotypes with known receptors and determined 3D structures of viral capsids. The validation dataset comprised 30 serotypes with known receptors but undetermined 3D structures of viral capsids. The prediction dataset consisted of 56 serotypes with unknown receptors but known genomic sequences (Tables S1–S3).

Based on whether uncoating receptors were identical, enterovirus serotypes within the datasets were paired up to form serotype pairs. Serotype pairs utilizing the same receptor were designated as “same-receptor serotype pairs”, while those utilizing different receptors were defined as “different-receptor serotype pairs” (Figure 2C). For example, CVB1 and CVB3 both utilize CAR as their uncoating receptor and therefore constitute a “same-receptor serotype pair”. In contrast, CVB1 and E6 employ CAR and FcRn, respectively, as their uncoating receptors and thus constitute a “different-receptor serotype pair”.

### 2.2. Sequences and Structures Analysis

The VP1–VP3 amino acid sequences of prototype strains from 104 enterovirus serotypes with determined genomic sequences were downloaded from the NCBI Nucleotide database [26] (https://www.ncbi.nlm.nih.gov/nuccore, accessed on 10 September 2025) (Tables S1–S3). The 3D structures of serotypes with determined viral capsid structures were downloaded from the RCSB Protein Data Bank (PDB) database [27] (https://www.rcsb.org), which were used to illustrate structural differences at receptor-binding sites, as well as to annotate secondary structure information (Figure 1). Multiple sequence alignments of the amino acid sequences were performed using the MUSCLE tool in MEGA 11 [28]. The PDB files and amino acid sequences were subsequently imported into the online tool ESPript 3.0 [29] (https://espript.ibcp.fr, accessed on 15 September 2025) to annotate secondary structure information.

### 2.3. Feature Selection

The model features comprised two categories: the amino acid sequences at receptor-binding sites and the physicochemical properties of amino acid residues (Figure 2B). These two types of features collectively determine the 3D structure of receptor-binding sites. Except for EVD, representative serotypes from other enterovirus species have had their receptor-binding sites experimentally determined. We collected the receptor-binding sites of these serotypes [9,14,15,16,17,18,19,20,21] and selected four physicochemical properties of amino acids (hydrophobicity, volume, surface accessibility, and polarity). These physicochemical properties are closely related to the 3D structure of the viral capsid; notably, Du et al. [30] constructed a predictive model based on these properties for identifying dominant viral strains to recommend influenza vaccines. Specific property values for the 20 amino acids were obtained from the AAIndex database [31] (https://www.genome.jp/aaindex, accessed on 15 September 2025) (Table S4). The physicochemical property values for amino acids in the AAindex database are predominantly relative indices, and not absolute physical quantities. These are derived from experimental or statistical data through standardization processes such as centering, normalization, or scaling. These indices are intended to reflect the relative differences among amino acids, as exemplified by hydrophobicity.

### 2.4. Prediction Algorithm

The fundamental principle of the prediction algorithm was to calculate the receptor similarity between enterovirus serotype pairs based on the conservation of receptor-binding sites and differences in physicochemical properties, and then to cluster the serotypes into groups using the network analysis method: serotypes utilizing the same receptor were grouped into the same cluster, while those utilizing different receptors were distributed across different clusters. Through this network clustering method, receptors for serotypes with unknown receptor usage were predicted, such that serotypes within the same cluster were inferred to utilize the same receptor (Figure 2C).

**Calculate the feature differences between serotype pairs.** The target variable Y represented receptor similarity, where 0 and 1 indicated that the serotype pair had “same receptor” and “different receptors”, respectively. For a given serotype pair, the receptor similarity was denoted as Yi (i = 1, …, N, where N was the total number of serotype pairs). The difference in the j-th feature (j = 1, …, m) for the i-th serotype pair was denoted as xij. The calculation of xij was defined as follows: for receptor-binding sites, xij = 0 if the amino acid residues of two serotypes were identical; otherwise, xij = 1. For physicochemical properties, xij was calculated as the average difference in feature values across all receptor-binding sites [30]. To prevent model overfitting, the feature differences of physicochemical properties were further discretized. To maximize the distinction of the target variable Y, a cutoff value for the feature difference of each physicochemical property was determined using the Receiver Operating Characteristic (ROC) curve analysis. Specifically, the cutoff value was calculated when the area under the ROC curve was maximized, which corresponded to the maximum Youden index. Based on this cutoff value, xij was converted into a binary variable (0 or 1).

For example, when calculating feature differences at receptor-binding sites between two serotypes, EV71 and CVA16 both possess the amino acid residue glycine (G) at VP2-137, while CVA10 contains serine (S). Thus, for the EV71-CVA16 serotype pair, xij = 0, and for the EV71-CVA10 serotype pair, xij = 1. When calculating differences in hydrophobicity between two serotypes at VP2-137, the hydrophobicity value of residue “G” is 0, and that of “S” is 1.7 (Table S4). Therefore, for the EV71-CVA16 serotype pair, xij = 0, and for the EV71-CVA10 serotype pair, xij = 1.7 (the absolute value).

**Build a Naive Bayes model.** This study constructed a Naive Bayes model to calculate receptor similarity. The Naive Bayes model is a machine learning method that is widely applied to classification problems in biological research. The conditional probability $P(Y|X_{1},\ldots ,X_{m})$ represents the probability that a serotype pair share the same receptor (Y = 0) or utilize different receptors (Y = 1), given the amino acid sequences and physicochemical properties of the receptor-binding sites. According to Bayesian theory:

(1) $$P(Y|X_{1},\ldots ,X_{m})=\frac{P(Y)\prod_{j=1}^{m}P(X_{j}|Y)}{P(X_{1},\ldots ,X_{m})}$$

The odds ratio (OR) was further defined to quantify the receptor similarity between serotype pairs. The OR was calculated as follows:

(2) $$OR=\frac{P(Y=0|X_{1},\ldots ,X_{m})}{P(Y=1|X_{1},\ldots ,X_{m})}=\frac{P(Y=0)}{P(Y=1)}\prod_{j=1}^{m}\frac{P(X_{j}|Y=0)}{P(X_{j}|Y=1)}$$

To avoid the occurrence of zero probabilities, Laplace smoothing coefficients were introduced for the probabilities (prior probability and conditional probability) on the right-hand side of Equation (2):

(3) $$P(Y=0)=\frac{1+\sum_{yi=0}1}{2+N}$$

(4) $$P(X_{j}|Y=0)=\frac{1+\sum_{yi=0\mathrm{and}xij=Xj}1}{2+\sum_{yi=0}1}$$

Through the aforementioned calculation method, the OR value can be obtained using Equation (2). When OR > 1, the two serotypes were considered to have similar receptors; a larger OR value indicated a higher probability that the two serotypes utilized the same receptor. Conversely, they had dissimilar receptors. It should be noted that the Naive Bayes model did not directly determine whether the receptors were identical; instead, it provided a measure of receptor similarity through the OR value. The determination of whether receptors were the same and the prediction of receptors were based on the similarity across all serotype pairs.

**Construct a receptor similarity network.** Based on the receptor similarity among serotype pairs, a receptor similarity network was constructed using Cytoscape 3.9 software [32]. In this network, nodes represent individual serotypes, and the distance between nodes reflects their receptor similarity (with distance defined as the reciprocal of lnOR). Shorter distances indicate greater receptor similarity. Finally, the network was partitioned into several clusters using the Markov Cluster (MCL) algorithm [33]. Within each cluster, the distances between serotypes were relatively short, indicating a high degree of receptor similarity and suggesting that these serotypes likely utilized the same receptor. In contrast, the distances between clusters were relatively long (with longer distances being obscured after clustering), indicating very low receptor similarity and suggesting that serotypes in different clusters likely utilized different receptors. Therefore, based on the known receptors in the training dataset, receptors of serotypes in the validation and prediction datasets can be predicted.

## 3. Results

### 3.1. Model Datasets and Serotype Pairs

There were a total of 104 human enterovirus serotypes with determined genomic sequences. These serotypes were divided into a training dataset, a validation dataset, and a prediction dataset (Figure 2A, Tables S1–S3). The 48 serotypes with known uncoating receptors were further categorized into training and validation datasets. Specifically, the training dataset, previously described by Wang et al. [9], consisted of 18 serotypes with determined 3D viral capsid structures, while the validation dataset comprised 30 serotypes lacking determined 3D structures. The remaining 56 serotypes with unknown receptors constituted the prediction dataset.

All serotypes within each dataset were systematically paired. Based on whether the receptors were identical, enterovirus serotypes were paired to generate serotype pairs. Serotype pairs sharing the same receptor were designated as “same-receptor serotype pairs”, whereas those utilizing different receptors were termed “different-receptor serotype pairs” (Table S5). Consequently, the 18 serotypes in the training dataset yielded 29 same-receptor serotype pairs and 124 different-receptor serotype pairs. Similarly, serotype pairs in the validation dataset were classified into the same two categories. However, for the subsequent network-based clustering analysis, the training and validation datasets were merged to create a combined set of serotype pairs. In total, the merged training and validation datasets yielded 257 same-receptor serotype pairs and 871 different-receptor serotype pairs (Figure 2C).

### 3.2. Binding Sites of Enterovirus Uncoating Receptors

In the training dataset, the 3D structures of virus-uncoating receptor binding complexes were determined for 10 serotypes, while the remaining eight were closely related serotypes. The receptor-binding sites for these 10 serotypes were collected from the corresponding literature [9,14,15,16,17,18,19,20,21] (Table S6). The binding sites of SCARB2 with EV71 were located on the VP1 GH loop and VP2 EF loop; the binding sites of KREMEN1 with CVA10 were located on the VP1 EF loop, GH loop, HI loop, and C-terminus, VP2 EF loop, VP3 BC loop, GH loop, and C-terminus; the binding sites of CAR with CVB3/CVB1 were primarily located on the VP1 BC loop, EF loop, GH loop, VP2 EF loop, and VP3 GH loop; the binding sites of FcRn with E6/E30 were mainly on the VP1 BC loop, EF loop, GH loop, and C-terminus, VP2 EF loop, and VP3 C-terminus; the binding sites of PV1-3 with CD155 were on the VP1 BC loop, EF loop, and GH loop, as well as the VP2 EF loop; and the binding sites of CVA24 with ICAM-1 were on the VP1 EF loop and GH loop, along with the VP2 EF loop. It could be observed that the VP1 BC, EF, GH loops and C-terminus, together with the VP2 EF loop, constituted the common major receptor-binding regions, all of which were located within the canyon and on its northern and southern walls.

The MUSCLE tool was used to perform multiple sequence alignment of the enterovirus VP1–VP3 amino acid sequences and to annotate the receptor-binding sites. After removing duplicate sites (where at least two receptors bound to the same site), a total of 104 uncoating receptor-binding sites were obtained (Figure S1). It was found that there indeed existed single receptor-binding sites capable of distinguishing different enterovirus species. For example, at VP1-229, EVA only had M, EVC all had D, and in EVB, except for E11, which had H, the rest all had N. At VP3-95, EVA all had P, EVB all had V, and EVC all had R; at VP1-160, EVB all had G, while EVA and EVC all had A; at VP3-91, EVC all had A, and EVA and EVB all had G. However, it was difficult to distinguish serotypes utilizing different receptors by a single site or simple combinations of sites. In addition, receptor-binding regions located in the loop regions of enteroviruses, such as the VP1 C-terminus and VP2 EF loop, exhibited high sequence variability but retained relatively conserved 3D structures (Figure 1C). Therefore, we hypothesized that the characteristics of receptor-binding sites could distinguish serotypes utilizing different receptors and were closely associated with their 3D structural features. However, this discrimination was not determined solely by sequence conservation but instead by a combination of sequence and physicochemical properties.

### 3.3. Model Prediction Results

**Figure 3:**
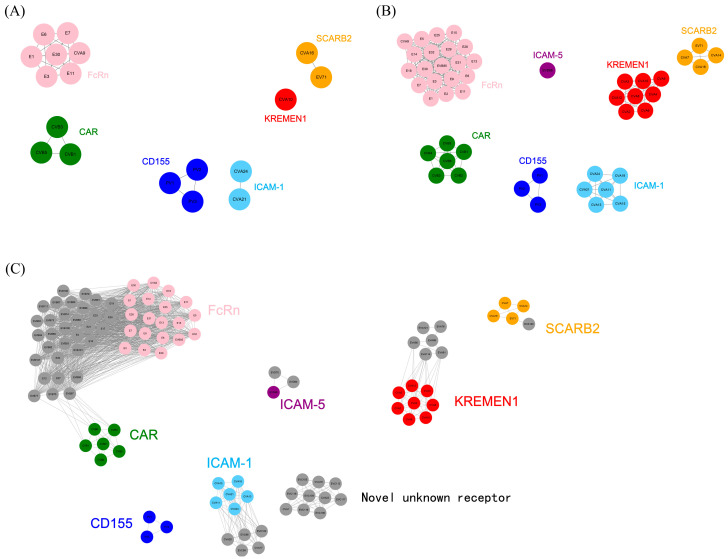
Prediction results of receptor usage in the training, validation and prediction datasets. (**A**–**C**) Model prediction results for the training, validation and prediction datasets. For ease of comparison, serotypes utilizing the same receptor were marked with the same color. (**A**) Prediction results for the training dataset. The 18 serotypes were clustered into six receptor-usage clusters. (**B**) Prediction results for the validation dataset. The 48 serotypes were clustered into seven receptor-usage clusters, whereas EVD68 utilized a distinct receptor, ICAM-5. (**C**) Prediction results for the prediction dataset. The 104 serotypes were clustered into eight receptor-usage clusters, among which 10 serotypes were predicted to utilize the same novel, unknown receptor.

The 18 serotypes in the training dataset were clustered into six receptor-usage clusters (Figure 3A, Table 1). EV71 and CVA16, which utilized the SCARB2 receptor, were grouped into a single cluster; CVA10, which utilized the KREMEN1 receptor, formed an independent cluster; CVB1, CVB3, and CVB5, which utilized the CAR receptor, were clustered together; CVA9 and echoviruses (E1, E3, E6, E7, E11, and E30) were grouped into a cluster utilizing the FcRn receptor; PV1-3 within EVC was clustered together, utilizing CD155 as the receptor; CVA21 and CVA24 were clustered together, with ICAM-1 serving as their receptor. Notably, all 18 serotypes in the training dataset were accurately classified according to the six known receptors.

Subsequently, the validation dataset was merged with the training dataset to construct a receptor similarity network, which was clustered into seven receptor-usage clusters and used to predict receptor usage for the validation dataset (Figure 3B, Table 1). In EVA, CVA7 and CVA14 were clustered together with EV71/CVA16 into a receptor-usage cluster utilizing SCARB2; CVA2, CVA3, CVA4, CVA5, CVA6, CVA8, and CVA12 were grouped together with CVA10 into a cluster utilizing the KREMEN1 receptor. In EVB, CVB (CVB1-6) was clustered into a single group utilizing the CAR receptor, while echoviruses were clustered together with CVA9 into another group utilizing the FcRn receptor. In EVC, CVA11, CVA13, CVA15, and CVA18 were grouped together with CVA21/CVA24 into a cluster utilizing the ICAM-1 receptor; only PV1-3 utilized the CD155 receptor. In EVD, EVD68 formed an independent cluster utilizing the ICAM-5 receptor. Notably, all serotypes in the validation dataset were correctly classified according to the seven receptors, with clear boundaries between receptor-usage clusters. These results demonstrate that the sequences of receptor-binding sites, together with their physicochemical properties, could reflect the underlying 3D structural features of receptor-binding sites and determine receptor usage. Because the receptor used by EVD68 (ICAM-5) differed from those represented in the training dataset, EVD68 did not cluster with the other six receptor-usage clusters but instead formed a separate cluster. This further demonstrated that the prediction algorithm could accurately classify receptor usage.

Finally, receptor usage was predicted for the 56 serotypes in the prediction dataset (Figure 3C, Table 1). The vast majority of these serotypes were predicted to utilize the seven known types of receptors. In EVA, EVA120 was predicted to utilize the SCARB2 receptor, while EVA76, EVA89, EVA90, EVA91, EVA114, and EVA121 were predicted to utilize the KREMEN1 receptor. All EVB serotypes in the prediction dataset were predicted to utilize the FcRn receptor. In EVC, CVA17, CVA20, EVC96, EVC99, and EVC102 were predicted to utilize the ICAM-1 receptor; CVA1, CVA19, CVA22, EVC104, EVC105, EVC109, EVC113, EVC116, EVC117, and EVC118 were predicted to utilize the same novel, unknown receptor. In EVD, EVD70 and EVD94 were predicted to utilize the ICAM-5 receptor. These predictions await experimental validation.

## 4. Discussion

### 4.1. Enterovirus-Induced Diseases and Functions of Receptors

Enterovirus infections can cause a wide spectrum of diseases: EVA primarily causes hand, foot, and mouth disease (HFMD) and herpangina [4,34]; EVB mainly causes HFMD, viral encephalitis, and myocarditis [3,35]; EVC is mainly responsible for poliomyelitis and acute flaccid paralysis [1,21]; and EVD predominantly induces acute flaccid myelitis, pneumonia, and bronchitis [2]. The major determinant underlying the disease diversity caused by different species or even different serotypes lies in the differences in viral receptor usage. Since receptor distribution exhibits tissue specificity, receptors confer tissue tropism to enteroviruses, and infection of different tissues results in divergent disease outcomes [8]. Enteroviruses utilize two classes of receptors: attachment receptors and uncoating receptors. Attachment receptors mediate the adsorption of enteroviruses to the cell surface. The binding of uncoating receptors to enteroviruses induces conformational changes in viral particles, triggering the release of lipid factors, externalization of the VP1 terminus, expulsion of VP4 from the capsids, and ultimately the release of viral genomes into the host cell cytoplasm [12,14]. Attachment receptors of enteroviruses are diverse, and individual serotypes may utilize multiple attachment receptors. For example, EV71 has been reported to use multiple attachment receptors, including P-selectin glycoprotein ligand-1 (PSGL-1), heparan sulfate (HS), annexin II (Anx2), sialic acid, and dendritic cell-specific ICAM3-grabbing non-integrin (DC-SIGN) [11,13,36]. EVD68 utilizes sialic acid and sulfated glycosaminoglycan (SGAG) as attachment receptors [37]. Additionally, attachment receptors are not restricted to specific viral serotypes. For instance, sialic acid serves as an attachment receptor for EV71, EVD68, and CVA24 [19,36,37]. Decay-accelerating factor (DAF) acts as an attachment receptor for numerous CVB serotypes and echoviruses within EVB [38].

Unlike attachment receptors, enteroviruses typically utilize a single, specific uncoating receptor. For example, although EV71 utilizes multiple attachment receptors, SCARB2 is its sole identified uncoating receptor. Receptor-mediated uncoating is a prerequisite for productive enterovirus infection, underscoring the critical role of uncoating receptors in viral entry.

### 4.2. From 3D Structure-Based Classification to Sequence-Based Receptor Prediction

To date, seven uncoating receptors of enteroviruses have been reported: SCARB2, KREMEN1, CAR, FcRn, ICAM-1, CD155, and ICAM-5. However, whether ICAM-5 functions as an uncoating receptor or merely as an attachment receptor for EVD68 remains inconclusive. The 3D structures of the first six receptors in complex with representative serotypes have been determined by cryo-electron microscopy (cryo-EM) (Table S6). These structures reveal that most receptors bind within the viral canyon, with only SCARB2 binding to the south puff region outside the canyon. Based on these observations, Wang et al. [9] proposed that viruses sharing the same uncoating receptor should display similar 3D structures at their receptor-binding regions. They successfully classified 18 serotypes into six receptor groups by clustering the 3D structural information of the VP1 EF and GH loops, as well as the VP2 EF loop. However, this algorithm was limited to viruses with experimentally determined 3D structures. Currently, high-resolution 3D structures have been determined for only 19 enteroviruses, including EVD68. To overcome this limitation, we explored the prediction of viral receptors based on viral amino acid sequences. We hypothesized that the amino acid sequences and physicochemical properties of receptor-binding sites could capture the corresponding 3D structural information. In other words, the 3D structural features at receptor-binding sites of enteroviruses utilizing different receptors could be reflected by amino acid sequences and physicochemical properties. Therefore, viral uncoating receptors could be predicted solely based on amino acid sequences.

In this study, we relied exclusively on amino acid sequences to predict the enterovirus receptor usage. The 3D structures of enteroviruses were used only to illustrate the structural differences at receptor-binding sites and to annotate secondary structure information. It should be noted that EVD68, the only known enterovirus in EVD reported to have an uncoating receptor, lacks the 3D structure of a virus-uncoating receptor complex, and its receptor-binding sites remain undefined. Moreover, because ICAM-5 has not been universally recognized as an uncoating receptor for EVD68, this serotype was included in the validation dataset. Among the 18 serotypes in the training dataset, 3D structures of virus-uncoating receptor complexes had only been determined for 10 serotypes. For the remaining eight serotypes, virus-receptor complexes were unavailable; however, the receptor-binding sites of their closely related serotypes were determined, enabling the assumption that their receptor-binding sites were similar to those of closely related serotypes. For example, CVA16 is closely related to EV71, and both utilize SCARB2 as the receptor; similarly, CVB5 is closely related to CVB1 and CVB3, all of which utilize CAR as the receptor. Therefore, the receptor-binding sites for all serotypes in the training dataset can be reasonably considered as determined.

### 4.3. Characteristics of Receptor-Binding Sites and Algorithm Development

The VP1 BC loop, EF loop, GH loop, C-terminus, and VP2 EF loop harbored the most extensively distributed receptor-binding sites (Figure S1). Multiple sites on the βE strand at the N-side of the VP2 EF loop displayed the species-specificity of enteroviruses. For instance, the conserved residues at position 125 were A, V, and F for EVA, EVB, and EVC, respectively. Additionally, VP2-123, 127, and 131-133 also displayed species-specific features. However, the receptor-binding region of the VP2 EF loop was highly variable in sequence, lacking a single site that determined species-specificity, let alone a simple single site or combination of sites that directly dictated receptor specificity. This observation highlighted the complexity of characteristics for receptor-binding sites. Based on the predictions of Wang et al. [9] and the 3D structures (Figure 1C), it was indeed observed that serotypes sharing the same receptor exhibited conservation in the 3D structure at receptor-binding sites. This strongly suggests that receptor-binding sites possess similar physicochemical properties. Although the sequences had changed, their physicochemical properties remained largely unchanged; otherwise, it would be challenging to maintain the conservation of the 3D structure. Therefore, we developed a bioinformatics algorithm based on amino acid sequences and their physicochemical properties to predict enterovirus receptors.

### 4.4. Challenges and Innovativeness of Prediction Algorithm

The prediction algorithm faces two challenges. The first challenge arises from the large number of receptor-binding sites (104 in total). Directly using all sites as features in a machine learning model would lead to the high dimensionality of feature variables. Moreover, some sites are completely conserved, while others are conserved only in certain serotypes, thus containing relatively low amounts of classification information. The second challenge lies in the large number of classification categories in the dataset, with the training dataset encompassing six classes and seven in the validation dataset. To address these two challenges, we first transformed the receptor classification task into a problem of determining receptor similarity between serotype pairs, discerning whether serotype pairs shared similar receptors. This approach transformed the feature variables from the receptor-binding site sequences and physicochemical properties to the differences in sequences and physicochemical properties at the receptor-binding sites. It significantly condensed the information of feature variables and amplified the feature differences between serotypes. Second, the prediction algorithm did not employ direct classification methods such as discriminant analysis, decision trees, or neural networks. Instead, we adopted a two-stage algorithm. In the first stage, the Bayesian method was used to calculate the receptor similarity between serotype pairs. This stage did not directly classify the receptors but provided information on which serotypes shared similar receptors with a given serotype. In the second stage, receptor classification clusters were determined through bioinformatic network analysis. In this way, serotypes utilizing the same receptor should cluster within the same group, and serotypes with unknown receptors could be predicted based on other known receptor-using serotypes within the same cluster. This predictive approach was inspired by the research on antigenic differences in influenza viruses [30]. From the results, it was evident that the algorithm accurately classified both the training and validation datasets and successfully predicted the prediction dataset. However, the term “prediction algorithm” here is more accurately described as a “classification algorithm”, as it assigns enterovirus serotypes to one of seven known receptor classes or to an unknown category. Nevertheless, the algorithm cannot predict the specific protein of unknown receptors, which constitutes a limitation of this study.

Numerous CVA serotypes could be grouped into four clusters, utilizing SCARB2, KREMEN1, FcRn, and ICAM-1 as the uncoating receptors, respectively. Additionally, CVB serotypes utilize CAR as their uncoating receptor. Overall, Coxsackieviruses utilize five distinct uncoating receptors, which largely account for their ability to infect a wide range of organs and tissues.

## 5. Conclusions

In conclusion, this study indicates that uncoating receptors of human enteroviruses can be accurately predicted based on the amino acid sequences and their physicochemical properties. Furthermore, the three-dimensional structural features of receptor-binding sites can be reflected through corresponding amino acid sequences and their physicochemical properties. This study facilitates a more in-depth investigation of enterovirus pathogenic mechanisms and provides important insights for the development of vaccines and antiviral drugs.

## Figures and Tables

**Table 1:** Prediction results of receptor usage in the training, validation and prediction datasets.

Species	Receptor-Usage Cluster	Training Dataset (18 Serotypes)	Validation Dataset (30 Serotypes)	Prediction Dataset (56 Serotypes)
EVA	SCARB2	EV71, CVA16	CVA7, CVA14	EVA120
	KREMEN1	CVA10	CVA2-6, CVA8, CVA12	EVA76, EVA89–91, EVA114, EVA121
EVB	CAR	CVB1, CVB3, CVB5	CVB2, CVB4, CVB6	
	FcRn	CVA9, E1, E3, E6, E7, E11, E30	E2, E5, E9, E13–15, E18, E25, E26, E29, E31, E32, EVB85	E4, E12, E16, E17, E19–21, E24, E27, E33, EVB69, EVB73–75, EVB77, EVB79–84, EVB86–88, EVB93, EVB97, EVB98, EVB100, EVB101, EVB106, EVB107, EVB111
EVC	CD155	PV1-3		
	ICAM-1	CVA21, CVA24	CVA11, CVA13, CVA15, CVA18	CVA17, CVA20, EVC96, EVC99, EVC102
EVD	ICAM-5		EVD68	EVD70, EVD94
	Novel unknown receptor			CVA1, CVA19, CVA22, EVC104, EVC105, EVC109, EVC113, EVC116–118

## Data Availability

The data presented in this study are available on request from the corresponding author.

## Supplementary Materials

The following supporting information can be downloaded at: https://www.mdpi.com/article/10.3390/v18020236/s1, Figure S1: Sequence alignment results of binding sites for uncoating receptors of enteroviruses; Table S1: Training dataset for the prediction model (18 serotypes); Table S2: Validation dataset for the prediction model (30 serotypes); Table S3: Prediction dataset for the prediction model (56 serotypes); Table S4: Physicochemical properties of 20 amino acids; Table S5: Serotype pairs composed of the training dataset (18 serotypes); Table S6: Specific binding sites of enterovirus uncoating receptors.

## Abbreviations

The following abbreviations are used in this manuscript:

3D	Three-dimensional
EVA	Enterovirus A
EVB	Enterovirus B
EVC	Enterovirus C
EVD	Enterovirus D
EV71	Enterovirus 71
CV	Coxsackievirus
E	Echovirus
PV	Poliovirus
HFMD	Hand, foot, and mouth disease
VP	Viral protein
SCARB2	Scavenger receptor class B, member 2
KREMEN1	Kringle containing transmembrane protein 1
CAR	Coxsackievirus and adenovirus receptor
FcRn	Human neonatal Fc receptor
ICAM-1	Intercellular adhesion molecule 1
CD155	Cluster of differentiation 155
PVR	Poliovirus receptor
ICAM-5	Intercellular adhesion molecule 5
ROC	Receiver operating characteristic
OR	Odds ratio
MCL	Markov cluster
PSGL-1	P-selectin glycoprotein ligand-1
HS	Heparan sulfate
Anx2	Annexin II
DC-SIGN	Dendritic cell-specific ICAM3-grabbing non-integrin
SGAG	Sialic acid and sulfated glycosaminoglycan
DAF	Decay-accelerating factor
cryo-EM	Cryo-electron microscopy
