# Supplementary material for: A Prediction Model for Uncoating Receptor Usage in Human Enteroviruses Based on Amino Acid Sequences and a Naive Bayes Algorithm
Source: Viruses. 2026 Feb 13;18(2):236. doi: 10.3390/v18020236 (PMC12944942; doi:10.3390/v18020236)
Supplement: Supplementary file 1 [file viruses-18-00236-s001.zip › Supplementary Files_Figure S1.pdf]

Supplementary Files: Figure S1

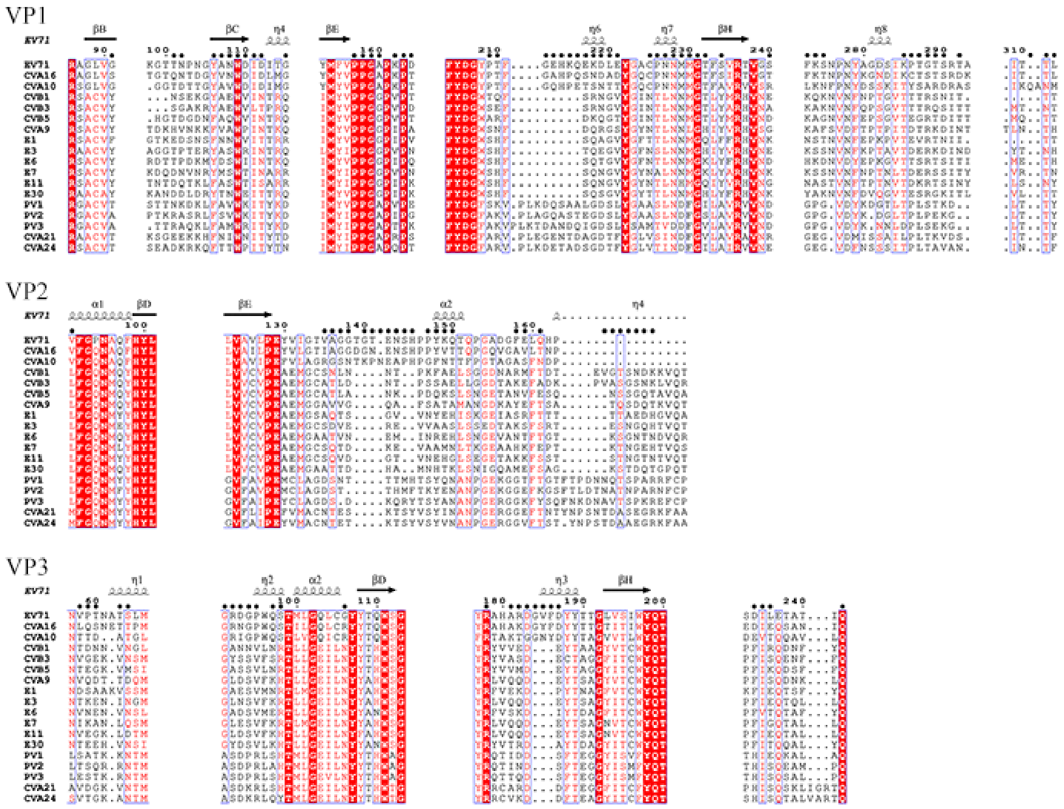

Figure S1. Sequence alignment results of binding sites for uncoating receptors of enteroviruses. The secondary structural regions of the amino acid sequences of enterovirus VP1-VP3 were intercepted. These regions contained 104 receptor-binding sites, which were marked with solid black dots.
